# Monitoring of Adverse Events in Recipients of the 2-Dose Ebola Vaccine Regimen of Ad26.ZEBOV Followed by MVA-BN-Filo in the UMURINZI Ebola Vaccination Campaign

**DOI:** 10.1093/infdis/jiac283

**Published:** 2022-07-01

**Authors:** Julien Nyombayire, Rosine Ingabire, Ben Magod, Amelia Mazzei, Jean-Baptiste Mazarati, Jozef Noben, Michael Katwere, Rachel Parker, Sabin Nsanzimana, Kristin M Wall, Felix Sayinzoga, Amanda Tichacek, Cynthia Robinson, Niina Hammoud, Frances Priddy, Susan Allen, Etienne Karita

**Affiliations:** Center for Family Health Research, Kigali, Rwanda; Center for Family Health Research, Kigali, Rwanda; Rwanda Zambia Health Research Group, Department of Pathology and Laboratory Medicine, School of Medicine, Emory University, Atlanta, Georgia, USA; Center for Family Health Research, Kigali, Rwanda; Department of Pathology, School of Medicine, Emory University, Atlanta, Georgia, USA; Rwanda Biomedical Center, Kigali, Rwanda; Janssen Global Public Health R&D, Beerse, Belgium; Janssen Vaccines and Prevention, Leiden, The Netherlands; Rwanda Zambia Health Research Group, Department of Pathology and Laboratory Medicine, School of Medicine, Emory University, Atlanta, Georgia, USA; Rwanda Biomedical Center, Kigali, Rwanda; Rwanda Zambia Health Research Group, Department of Pathology and Laboratory Medicine, School of Medicine, Emory University, Atlanta, Georgia, USA; Department of Epidemiology, Rollins School of Public Health, Emory University, Atlanta, Georgia, USA; Rwanda Biomedical Center, Kigali, Rwanda; Rwanda Zambia Health Research Group, Department of Pathology and Laboratory Medicine, School of Medicine, Emory University, Atlanta, Georgia, USA; Janssen Vaccines and Prevention, Leiden, The Netherlands; Janssen Vaccines and Prevention, Leiden, The Netherlands; Independent Consultant, Wellington, New Zealand; Rwanda Zambia Health Research Group, Department of Pathology and Laboratory Medicine, School of Medicine, Emory University, Atlanta, Georgia, USA; Department of Pathology, School of Medicine, Emory University, Atlanta, Georgia, USA

**Keywords:** Ebola, vaccination, unsolicited adverse events, serious adverse events, Rwanda

## Abstract

**Background:**

From 2019 to 2021, Rwandan residents of the border with the Democratic Republic of the Congo were offered the Ad26.ZEBOV (adenovirus type 26 vector vaccine encoding Ebola virus glycoprotein) and MVA-BN-Filo (modified vaccinia virus Ankara vector vaccine, encoding glycoproteins from Ebola, Sudan, Marburg, and nucleoprotein from Tai Forest viruses) Ebola vaccine regimen.

**Methods:**

Nonpregnant persons aged ≥2 years were eligible. Unsolicited adverse events (UAEs) were reported through phone calls or visits, and serious adverse events (SAEs) were recorded per International Council for Harmonisation of Technical Requirements for Pharmaceuticals for Human Use guidelines.

**Results:**

Following Ad26.ZEBOV, UAEs were reported by 0.68% of 216 113 vaccinees and were more common in younger children (aged 2–8 years, 1.2%) compared with older children (aged 9–17 years, 0.4%) and adults (aged ≥18 years, 0.7%). Fever and headache were the most reported symptoms. All 17 SAEs related to vaccine were in children aged 2–8 years (10 postvaccination febrile convulsions ± gastroenteritis and 7 fever and/or gastroenteritis). The incidence of febrile seizures was 8 of 26 062 (0.031%) prior to initiation of routine acetaminophen in December 2020 and 2 of 15 897 (0.013%) thereafter. Nonobstetric SAEs were similar in males and females. All 20 deaths were unrelated to vaccination. Young girls and adult women with UAEs were less likely to receive the second dose than those without UAEs. Seven unrelated SAEs occurred in 203 267 MVA-BN-Filo recipients.

**Conclusions:**

Postvaccination febrile convulsions in young children were rare but not previously described after Ad26.ZEBOV and were reduced with routine acetaminophen. The regimen was otherwise safe and well-tolerated.

## Teaser

UMURINZI, the largest campaign of the Ad26.ZEBOV/MVA-BN-Filo Ebola vaccination, confirmed previous trials’ safety conclusions. Notable findings were rare febrile seizures in young children, reduced second-dose retention in women with incident pregnancy, and more obstetric vs nonobstetric serious adverse events.

Since the first known outbreak of Ebola virus in 1976, there have been roughly 29 outbreaks of the virus [1]. The 2013–2016 West African Ebola epidemic, the largest outbreak, led to rapid progression of research, prevention strategies, and vaccine development. One of the vaccines to emerge is the heterologous 2-dose vaccination regimen of the adenovirus type 26 vector vaccine encoding Ebola virus glycoprotein (Ad26.ZEBOV) and the modified vaccinia virus Ankara vector vaccine, encoding glycoproteins from Ebola, Sudan, Marburg, and nucleoprotein from Tai Forest viruses (MVA-BN-Filo), developed by Janssen Vaccines & Prevention B.V.

While the epidemic in West Africa brought the Ebola virus to the forefront of international attention, the Democratic Republic of the Congo (DRC) has been a more frequent hotspot for Ebola with 13 total outbreaks, the most of any country [2]. The 2018–2020 DRC epidemic began in the eastern border provinces of North Kivu and Ituri and spread to South Kivu. During this epidemic, the World Health Organization (WHO) declared it the highest level of emergency and escalated its response to recommend, in May 2019, that populations in the region be vaccinated with the Ad26.ZEBOV/MVA-BN-Filo Ebola vaccine regimen [3]. Further advice from the WHO encouraged at-risk countries to approve “investigational medicines and vaccines as an immediate priority for preparedness” [4].

On DRC’s eastern border, about 99 miles from the epicenter of the 2018 outbreak, that same year, a news article reported that 90 000 people sought passage across the Rwanda-DRC border each day, making this the second-busiest border crossing in the world [5]. With this context, in response to neighboring DRC struggling to contain the Ebola outbreak and the WHO recommendations, on 27 September 2019, the Rwandan Food and Drug Administration approved exceptional emergency use of the 2-dose Ad26.ZEBOV/MVA-BN-Filo Ebola vaccine regimen to protect Rwandan residents [6]. An initiative deemed the UMURINZI (“guardian” in the Kinyarwanda vernacular) Ebola Vaccination Campaign (Unprecedented Movement to drive a Unified Rwandan Initiative for National ZEBOVAC Immunization) was launched with the goal of delivering this vaccine regimen to 200 000 nonpregnant Rwandan residents in those bordering regions most at risk of Ebola spread from the DRC. Johnson & Johnson (J&J) donated the 200 000 vaccine doses and, along with the UK Department for International Development and the Wellcome Trust, provided funding for the campaign [7].

The heterologous 2-dose Ebola vaccine regimen, Ad26.ZEBOV followed by MVA-BN-Filo 56 days later, has been shown to be immunogenic, well-tolerated, and safe in 45 phase 1, 12 phase 2, and 23 phase 3 clinical trials [8–15]. Several other studies are currently ongoing or under investigation. Across these studies, only a few thousand individuals have been vaccinated, with 54 participants reporting serious adverse events (SAEs) (2.8%). Of those SAEs, there were no specific safety concerns raised, and only 1 case was deemed possibly related to the vaccine. While prior data show a reassuring safety profile and low rate of SAEs, this vaccine regimen has not previously been utilized in a preventive vaccination approach or studied in the Rwandan population. Given that the UMURINZI Ebola Vaccination Campaign is the first utilization of this vaccine in such a large population, we present reported signs and symptoms in patient-initiated calls and clinic visits and the subset of those that are SAEs in those who have received the first dose, the Ad26.ZEBOV vaccination. The unsolicited adverse events (UAEs)/SAEs reported after the second dose are described, though briefly, given small numbers. This large UMURINZI campaign will contribute to what has been learned from prior smaller studies and programs.

## METHODS

Inclusion criteria for vaccination in the UMURINZI campaign were Rwandan citizens ≥2 years of age and residing in the districts bordering DRC. Participants were excluded if they had any current or chronic serious illness or if pregnant per a urine pregnancy test.

Fifteen vaccination sites were launched including 8 sites in Rubavu district, on the northwestern border of Rwanda across the border from Goma, the capital city of North Kivu, DRC, and <100 miles from Ituri, ground zero for the 2018 outbreak. Six other vaccination sites were located in the southwestern Rwandan border district of Rusizi across the border from Bukavu, the capital city of South Kivu, DRC. One additional vaccination site was in Kigali, the capital city of Rwanda, for residents who traveled routinely to the DRC border area.

UAEs and SAEs postvaccination with the Ad26.ZEBOVdose but prior to MVA-BN-Filo vaccination were included. There were 7 SAEs reported after MVA-BN-Filo vaccination; these are briefly described but excluded from further detailed analysis due to small numbers. UAEs were reported by participants and family members by phone or in person at vaccination sites and local healthcare facilities. SAEs were investigated by UMURINZI physicians who were often involved in monitoring hospitalizations. An Adverse Event Following Immunization Reporting Form created by and for submission to the Rwanda Food and Drug Administration and a Global Complaint Data Collection Form created by and for submission to Janssen Pharmaceuticals were completed. As determined by these forms, designation of an adverse event as an SAE required death, permanent/significant disability, life-threatening, congenital anomaly/birth defect, or hospitalization. SAEs resulting from poor pregnancy outcomes (miscarriage, stillbirth, etc, none related to vaccination) are described in detail in a separate manuscript and are not included here. Collected data included demographics, vaccination date, SAE start and end dates, SAE medical narratives and diagnoses, and whether the second dose was administered. As part of these case reporting forms, a determination of the relationship of the event to the vaccine was made by the reporter and UMURINZI investigating physicians. The medical diagnoses/description of SAE terms characterizing each case were made by the investigating physician in correspondence with local healthcare professionals and medical records; some similar, synonymous, or overlapping diagnoses were combined for analysis.

Data were electronically captured in SurveyCTO (Dobility, Cambridge, Massachusetts) using the SurveyCTO Collect app on Android tablets and were exported from SurveyCTO into Microsoft Access for data cleaning. For analysis, cases were divided into early childhood (ages 2–8 years), middle childhood and adolescent (ages 9–17 years), and adult (≥18 years) populations. All postvaccination UAEs/SAEs prior to 31 October 2021, including calls and visits, were analyzed by age group and sex. SAS software version 9.4 (SAS Institute, Cary, North Carolina) was used to compare frequencies of symptoms between age groups using χ^2^ tests of significance. IBM SPSS (version 26, IBM Corporation, Armonk, New York) was used to perform descriptive statistics.

Verbal consent was obtained during the vaccination campaign, and those willing to provide contact and biometric data, to be vaccinated, and to have adverse events recorded were provided with the vaccine. A nonresearch determination for this Centers for Disease Control and Prevention–defined program was submitted to Emory University.

## RESULTS

From initiation and the first vaccination on 8 December 2019, the UMURINZI program vaccinated 216 113 individuals with the Ad26.ZEBOV dose (last dose 22 July 2021). The proportion of UAEs (calls or visits to UMURINZI or health centers) was highest at 1.2% in the 2- to 8-year-olds who constituted 19.4% of vaccinees; in contrast, only 0.4% of 9- to 17-year-olds (who constituted 29.4% of vaccinees), and 0.7% in those aged ≥18 years (the remaining 51.2% of vaccinated individuals) reported UAEs (Table 1). UAE symptoms included fever (65%), headache (51%), fatigue (21%), and chills (18%).

**Table 1.** Unsolicited and Serious Adverse Events After Ad26.ZEBOV Vaccination by Sex and Age Group, With Retention for Second Dose of MVA-BN-Filo

Age and Sex	All Vaccinees, No.	UAEs, No.	Proportion UAEs	SAEs, No.	Proportion SAEs/UAEs	Proportion SAEs/All Vaccinated
Male						
ȃ2–8 y	20 868	241	1.2%	10	4.1%	0.048%
ȃ9–17 y	30 236	105	0.3%	3	2.9%	0.010%
ȃ≥18 y	46 960	292	0.62%	12	4.1%	0.026%
Female						
ȃ2–8 y	21 091	251	1.2%	12	4.8%	0.057%
ȃ9–17 y	33 216	126	0.4%	7	5.6%	0.021%
ȃ≥18 y	63 742	328	0.71%	10	3.0%	0.016%
Age and Sex	All UAEs, No.	% Second Dose for Those With UAEs	% Second Dose for Non-UAE	*P* Value (χ^2^) UAE vs Non-UAE	SAEs, No.	% Second Dose for Those With SAEs
Male						
ȃ2–8 y	241	94%	96%	.060	10	60%
ȃ9–17 y	105	93%	94%	.620	3	0%
ȃ≥18 y	292	91%	91%	.979	12	25%
Female						
ȃ2–8 y	251	92%	97%	.0001	12	50%
ȃ9–17 y	126	93%	95%	.223	7	71%
ȃ≥18 y	328	86%	94%	<.0001	10	76%

Abbreviations: Ad26.ZEBOV, adenovirus type 26 vector vaccine encoding Ebola virus glycoprotein; MVA-BN-Filo, modified vaccinia virus Ankara vector vaccine, encoding glycoproteins from Ebola, Sudan, Marburg, and nucleoprotein from Tai Forest viruses; SAE, serious adverse event; UAE, unsolicited adverse event.

The proportion of vaccinees with SAEs was also highest in the youngest age group at 0.052%, compared to 0.016% in ages 9–17 and 0.020% in adults.

Retention for the second dose with MVA-BN-Filo vaccination was high with 203 267 (94%) returning a mean of 70 days (median, 57 days) after dose 1. The last dose of MVA-BN-Filo was given on 13 September 2021. The impact of reporting an UAE on the likelihood of returning for the second dose is shown in Table 1. Girls aged 2–8 years who reported UAEs were significantly less likely to receive the second dose compared to girls without UAEs (92% vs 97%, *P* = .0001). The trend was also seen in boys, though with borderline significance (94% vs 96%, *P* = .060). Males and females in the 9–17 age group were similar in their retention rates, and those with UAEs were no less likely to receive dose 2 than those without. Overall retention among adults was lower than among younger groups, and UAEs did not affect likelihood of receiving the second dose among men. Retention was lower among women than among men, in part due to pregnancy: 1.5% of women became pregnant between dose 1 and dose 2. While women were eligible for dose 2 postpartum, a good number of pregnancies were detected late enough in the program that vaccinations had stopped prior to the deliveries. As a result, among women who became pregnant after dose 1, only 41% received dose 2. UAEs and SAEs were considerably less frequent following dose 2 compared with dose 1. There were 159 UAEs after dose 2 compared with 1343 UAEs after dose 1. There were 178 SAEs after dose 1: 146 in adults, 10 in 9- to 17-year-olds, and 22 in 2- to 8-year-olds.

Table 2 presents the details of SAEs in 2- to 8-year-olds, ordered by deaths first (4, all unrelated), followed by unrelated (1) then related (17) SAEs. Age, sex, and days between vaccination and onset are shown. Boys and girls did not differ with respect to frequency or presentation of SAEs. The deaths were deemed unrelated based on the delay between vaccination and onset and/or the presence of another diagnosis. One death in a 3-year-old girl followed acute diarrhea and vomiting but detailed investigations confirmed that, though the death occurred the day after vaccination, the child expelled ascaris worms at the health center and the attending physician attributed cause of death to asphyxiation after vomiting. Similarly, a hospitalization for severe malaria in a 6-year-old boy occurred the night after vaccination but was confirmed by laboratory diagnosis with death occurring 26 days later despite therapy. A case of severe abdominal pain in a 6-year-old girl that began 3 days after vaccination resulted in death 24 days later after multiple interactions with health centers. All 17 cases considered related to Ad26.ZEBOV began the evening or day after the vaccination. The 10 cases that included febrile convulsions with or without fever and/or diarrhea responded to appropriate therapy with hospital discharge within 2 days, as did the 7 cases with fever and/or diarrhea/vomiting, with the longest hospitalization lasting 4 days.

**Table 2.** Serious Adverse Events After Ad26.ZEBOV Vaccination in Children Aged 2–8 Years

UMURINZI ID	Medical Diagnosis/Description of SAEs	Patient Sex	Patient Age, y	Ad26.ZEBOV Vx Date	SAE Start Date	SAE Start Date – Vx Date	SAE End Date/Date of Death When Applicable	SAE End Date/Death Date – Vx Date
R0056208	Death, vomiting, diarrhea, ascaris, asphyxiation due to vomiting	F	3	12 Jun 2020	13 Jun 2020	1	13 Jun 2020	1
R0009304	Death, severe malaria	M	6	15 Jan 2020	15 Jan 2020	0	11 Feb 2020	27
R0075449	Death, unknown cause, severe abdominal pain	F	6	5 Aug 2020	8 Aug 2020	3	1 Sep 2020	27
R0086565	Death, acute severe malnutrition and staphylococcal skin infection	F	2	8 Sep 2020	7 Dec 2020	90	30 Dec 2020	113
R0009419	Cerebral malaria	F	2	16 Jan 2020	16 Jan 2020	0	23 Jan 2020	7
R0025554	Post-Vx febrile convulsions	M	3	11 Mar 2020	11 Mar 2020	0	11 Mar 2020	0
R0068048	Post-Vx febrile convulsions	M	7	3 Jul 2020	3 Jul 2020	0	3 Jul 2020	0
R0136847	Post-Vx febrile convulsions	F	2	23 Dec 2020	23 Dec 2020	0	24 Dec 2020	1
R0044862	Post-Vx febrile convulsions	M	2	3 Nov 2020	3 Nov 2020	0	4 Nov 2020	1
R0128953	Post-Vx febrile convulsions	F	3	25 Nov 2020	25 Nov 2020	0	26 Nov 2020	1
R0104597	Post-Vx febrile convulsions	F	3	30 Sep 2020	30 Sep 2020	0	1 Oct 2020	1
R0162444	Post-Vx febrile convulsions	M	3	4 Feb 2021	4 Feb 2021	0	5 Feb 2021	1
R0119807	Post-Vx febrile convulsions	M	2	24 Nov 2020	24 Nov 2020	0	26 Nov 2020	2
R0105159	Post-Vx febrile convulsions, diarrhea, vomiting	M	3	28 Sep 2020	28 Sep 2020	0	29 Sep 2020	1
R0116808	Post-Vx febrile convulsions, vomiting	F	3	6 Nov 2020	6 Nov 2020	0	8 Nov 2020	2
R0109181	Post-Vx fever	F	3	9 Oct 2020	10 Oct 2020	1	11 Oct 2020	2
R0128179	Post-Vx fever	M	2	18 Dec 2020	18 Dec 2020	0	21 Dec 2020	3
R0064800	Post-Vx fever	F	2	15 Jul 2020	15 Jul 2020	0	19 Jul 2020	4
R0140682	Post-Vx fever, vomiting, diarrhea	M	3	15 Jan 2021	15 Jan 2021	0	16 Jan 2021	1
R0110662	Post-Vx fever, vomiting, diarrhea	F	2	29 Oct 2020	29 Oct 2020	0	31 Oct 2020	2
R0011670	Post-Vx weakness, vomiting	F	2	28 Jan 2020	28 Jan 2020	0	29 Jan 2020	1
R0028088	Post-Vx fever, diarrhea, vomiting, weakness	M	3	20 Mar 2020	20 Mar 2020	0	22 Mar 2020	2

Abbreviations: Ad26.ZEBOV, adenovirus type 26 vector vaccine encoding Ebola virus glycoprotein; F, female; M, male; SAE, serious adverse event; UMURINZI, Unprecedented Movement to drive a Unified Rwandan Initiative for National ZEBOVAC Immunization; Vx, vaccination.

Due to concerns about the reported cases of febrile convulsions in young vaccinees, the decision was made to institute routine acetaminophen administration via 250-mg suppository at the time of vaccination and again 6 hours later at home. This policy was implemented after the holiday closure in December 2020. Thereafter, 2 cases of febrile convulsions occurred in young children, neither of whom received the second acetaminophen suppository in the home. The incidence of febrile seizures was 8 of 26 062 (0.031%) prior to initiation of routine acetaminophen and 2 of 15 897 (0.013%) after.

There were 4 SAEs in the 9–17 years age group (Table 3), all resulting in death 11–139 days after vaccination and none considered related to vaccine.

**Table 3.** Serious Adverse Events After Ad26.ZEBOV in Youths Aged 9–17 Years

UMURINZI ID	Medical Diagnosis/Description of SAE	Patient Sex	Patient Age, y	Ad26.ZEBOV Vx Date	SAE Start Date	Date SAE – Vx Date	Date of Death	SAE End Date/Death Date – Vx Date
R0148332	Death, head and abdominal pain	M	9	14 Jan 2021	24 Jan 2021	10	25 Jan 2021	11
R0125707	Death, severe sepsis with suspected leukemia	F	11	18 Dec 2020	16 Jan 2021	29	31 Jan 2021	44
R0035649	Death, crushing trauma	M	11	27 May 2020	13 Oct 2020	139	13 Oct 2020	139
R0088778	Death, food poisoning	M	12	8 Sep 2020	22 Nov 2020	75	24 Nov 2020	77

Abbreviations: Ad26.ZEBOV, adenovirus type 26 vector vaccine encoding Ebola virus glycoprotein; F, female; M, male; SAE, serious adverse event; UMURINZI, Unprecedented Movement to drive a Unified Rwandan Initiative for National ZEBOVAC Immunization; Vx, vaccination.

SAEs in adults are presented in Table 4. There were 3 deaths in women 29–251 days after vaccination, none related to vaccine. There were 9 deaths in men 14–157 days after vaccination, again none related to vaccine. All deaths had either a clear cause of death and/or a prior history of similar problems. There were an additional 7 SAEs in women 68–296 days after vaccination. Four were hospitalizations due to urinary tract infections, 3 occurring in pregnant women including 2 cases with vomiting. One woman became pregnant and developed deep vein thrombosis 296 days after vaccination. The last remaining 2 cases involved an episode of generalized arthralgia in a woman with a recurrent history of this problem, and 1 hospitalization for pharyngitis in a pregnant woman. Last, 2 hospitalizations occurred among men 2 and 41 days after vaccination, neither associated with vaccination.

**Table 4.** Serious Adverse Events After Ad26.ZEBOV in Adults Aged ≥18 Years

UMURINZI ID	Medical Diagnosis/Description of SAE	Patient Sex	Patient Age, y	Ad26.ZEBOV Vx Date	SAE Start Date	SAE End Date/Date of Death When Applicable	SAE Start Date – Vx Date	SAE End Date/Death Date – Vx Date
R0009924	Death, 5-y Hx of seizures and loss of consciousness	F	59	20 Jan 2020	15 Feb 2020	18 Feb 2020	26	29
R0024488	Death, long Hx of hypertension and diabetes, recent leg wound debridement, abdominal pain just prior to death	F	75	3 Mar 2020	28 May 2020	9 Jun 2020	86	98
R0040653	Death following delivery, COVID-19	F	33	5 Nov 2020	5 Jul 2021	14 Jul 2021	242	251
R0106609	Death, drowning	M	33	14 Oct 2020	28 Oct 2020	28 Oct 2020	14	14
R0116141	Death, COVID-19	M	23	16 Nov 2020	28 Nov 2020	2 Dec 2020	12	16
R0028273	Death, 4-y Hx of seizures and syncope	M	28	2 Jun 2020	19 Jun 2020	19 Jun 2020	17	17
R0013530	Death, Hx of peptic ulcer disease and hypertension	M	71	10 Feb 2020	2 Mar 2020	2 Mar 2020	21	21
R0092839	Death, post-Rx for gastritis and dry cough	M	56	2 Oct 2020	28 Oct 2020	1 Nov 2020	26	30
R0006487	Death, colon cancer	M	75	21 Jan 2020	27 Feb 2020	5 Mar 2020	37	44
R0100105	Death, road accident	M	25	14 Sep 2020	1 Nov 2020	1 Nov 2020	48	48
R0089844	Death, severe malaria	M	73	30 Sep 2020	23 Dec 2020	29 Dec 2020	84	90
R0023494	Death, cholangiocarcinoma	M	62	28 Feb 2020	5 Apr 2020	30 May 2020	37	92
R0008031	Death, gunshot wound	M	40	6 Jan 2020	11 Jun 2020	11 Jun 2020	157	157
R0020052	Pharyngitis in pregnancy	F	40	27 Feb 2020	5 May 2020	8 May 2020	68	71
R0024271	UTI	F	35	2 Mar 2020	11 May 2020	13 May 2020	70	72
R0065566	UTI and vomiting in pregnancy	F	28	21 Jul 2020	5 Oct 2020	7 Oct 2020	76	78
R0008155	UTI in pregnancy	F	31	7 Jan 2020	28 May 2020	5 Jun 2020	142	150
R0030218	UTI and vomiting in pregnancy	F	20	26 May 2020	20 Oct 2020	24 Oct 2020	147	151
R0005162	DVT in pregnancy	F	34	27 Dec 2019	18 Oct 2020	Unknown	296	…
R0001266	Generalized arthralgia with prior Hx	F	36	20 Dec 2019	5 Feb 2020	Ongoing	47	…
R0023248	Severe lower back pain	M	49	24 Feb 2020	26 Feb 2020	3 Mar 2020	2	8
R0006546	Seizure	M	62	16 Jan 2020	26 Feb 2020	28 Feb 2020	41	43

Abbreviations: Ad26.ZEBOV, adenovirus type 26 vector vaccine encoding Ebola virus glycoprotein; COVID-19, coronavirus disease 2019; DVT, deep vein thrombosis; F, female; Hx, history; M, male; Rx, prescription; SAE, serious adverse event; UMURINZI, Unprecedented Movement to drive a Unified Rwandan Initiative for National ZEBOVAC Immunization; UTI, urinary tract infection; Vx, vaccination.

Table 5 details the 7 SAEs that occurred 12–120 days after the second dose with MVA-BN-Filo, none of which were related to vaccination. Two deaths were due to tuberculosis confirmed with GeneXpert testing and suspected cholera, respectively. Two adult patients aged 51 and 67 years were hospitalized with hypertension, both stabilizing with treatment. One 6-year-old developed jaundice 4 days after MVA-BN-Filo administration and was diagnosed with cholestatic liver disease based on thickened gallbladder wall on ultrasound and elevated liver enzymes with negative hepatitis B testing.

**Table 5.** Serious Adverse Events After MVA-BN-Filo Vaccination

UMURINZI ID	Medical Diagnosis/Description of SAE	Patient Sex	Patient Age, y	Ad26.ZEBOV Vx Date	MVA-BN-Filo Vx Date	SAE Start Date	SAE End Date/Date of Death When Applicable	SAE Start Date – Vx Date	SAE End Date/Death Date – Vx Date
R0017927	Death, pulmonary tuberculosis	F	31	29 Jan 2020	6 May 2020	8 May 2020	18 May 2020	2	12
R0059693	Death, hypovolemic shock due to suspected cholera	M	12	26 Jun 2020	7 Sep 2020	24 Nov 2020	26 Nov 2020	78	80
R0001887	Hypertension	M	51	30 Dec 2019	24 Feb 2020	5 Mar 2020	9 Mar 2020	10	14
R0060933	Hypertension	F	67	23 Jun 2020	20 Aug 2020	26 Aug 2020	3 Sep 2020	6	14
R0083434	Suspected cholestatic liver disease	M	6	6 Aug 2020	1 Oct 2020	5 Oct 2020	Resolved^a^	4	…
R0009783	Spontaneous abortion	F	29	17 Jan 2020	13 Mar 2020	16 Mar 2020	11 Jun 2020	3	90
R0026834	Spontaneous abortion	F	30	17 Mar 2020	7 May 2020	2 Sep 2020	4 Sep 2020	118	120

Abbreviations: Ad26.ZEBOV, adenovirus type 26 vector vaccine encoding Ebola virus glycoprotein; F, female; M, male; MVA-BN-Filo, modified vaccinia virus Ankara vector vaccine, encoding glycoproteins from Ebola, Sudan, Marburg, and nucleoprotein from Tai Forest viruses; SAE, serious adverse event; UMURINZI, Unprecedented Movement to drive a Unified Rwandan Initiative for National ZEBOVAC Immunization; Vx, vaccination.

Unknown date, resolved as of 2021.

## DISCUSSION

The UMURINZI program is the largest vaccination campaign using the Ad26.ZEBOV and MVA-BN-Filo Ebola vaccination regimen to date and the first time the vaccine has been used in the Rwandan population. While prior studies in phase 1, 2, and 3 trials have concluded that this vaccination regimen is well-tolerated and safe, leading European and Rwandan drug safety organizations to approve its use, postmarketing data and larger sample sizes warrant investigation to further confirm safety conclusions and monitor for rarer events. The most notable finding in this campaign was the rare occurrence of febrile seizures in young children, which was reduced by routine postvaccination treatment with acetaminophen suppositories. Another important observation was the role incident pregnancy played in reducing retention for the second dose in women of childbearing age, and in the comparatively large number of obstetric SAEs (described in a separate manuscript) compared with nonobstetric SAEs in this group.

Despite pauses due to the coronavirus disease 2019 pandemic beginning in March 2020 and a volcanic eruption in Goma, DRC, in May 2021, 216 113 Rwandan individuals were vaccinated across 15 different sites in regions with high traffic and high risk of infectious disease spread from DRC. The retention was impressively high (94%) for this vaccination campaign, likely a product of engaged participants, diligent staff, and organizational, structural, and managerial planning, and strong support from local leaders. It is also helpful that Rwanda is a small country with 1 local language, paved roads between major cities, and free radio and television publicity for public health campaigns.

Overall, there were 178 reported SAEs after dose 1. Compared to prior clinical trial data where SAEs were reported in 2.8% of vaccinated participants and 2.4% of participants in the placebo groups, the current rate of SAE reports in the UMURINZI campaign is much lower at 0.04% of all vaccinated individuals [8–13, 15]. Although we did not place a time limit on postvaccination reporting of UAEs/SAEs other than the end of the vaccination campaign, this low rate is likely due to spontaneous reporting rather than systematic follow-up of all participants as has occurred in clinical trials. Despite the significant outreach, the involvement of local hospitals, health clinics, and community health workers, and participants’ high return to follow-up rates, some SAEs may have been missed.

Ten cases of postvaccination febrile convulsions were reported, all occurring in children aged 7 years or younger. These cases all occurred within 24 hours of vaccine administration; all subsequently recovered under medical care without sequelae within 48 hours. This SAE has not previously been described in prior study of this vaccine regimen including in pediatric participants. The UMURINZI campaign has vaccinated significantly more children than prior studies, likely contributing to detection of these rare events. Febrile seizures are common in children with as many as 5% of all children having a febrile seizure in their lifetime [16]. A survey of febrile seizures in Kenya noted that most were associated with malaria and respiratory infections, but that incidence estimates vary greatly [17]. Several other vaccines have been associated with increased risk of febrile seizures in children including mumps-measles-rubella (MMR) vaccine, DTP (diphtheria, tetanus, and pertussis) vaccine, inactivated influenza vaccine, and 13-valent pneumococcal conjugate vaccine in Western countries. In 1 study of the inactivated influenza vaccine in the United States, the risk of febrile seizure was 9 per 1000 doses in young children [18]. For the MMR vaccine, the rate of febrile seizures in children in England was evidenced to be 33 per 100 000 after vaccination [18–23]. History of past febrile convulsions was not collected prior to vaccination, nor was it an exclusion criterion in this campaign. However, this could be considered for future campaigns.

The incidence of convulsions following routine vaccination in Africa is difficult to find as SAEs are not systematically reported. One in-depth study in Kenya showed 3 of 457 infants having convulsions during the 14-week vaccination schedule, although which vaccine preceded the convulsions was not specified [24]. Passive surveillance following a mass campaign with meningococcal A vaccine in West Africa found that convulsions were noted in 0.032% of 11.5 million vaccinees but found a similar rate of convulsions in historical data unrelated to vaccine administration [25].

While febrile seizures related to vaccination are not uncommon in other vaccines, their incidence after the Ad26.ZEBOV vaccine had not previously been reported. The 10 cases of this SAE in early childhood vaccinees were concerning and led to institutional changes in this age group of participants in the UMURINZI campaign. As a result of the cases of postvaccination febrile convulsions, the UMURINZI medical staff, at the direction of the sponsor on 3 December 2020, instituted 1 dose of acetaminophen at the time of administration of the vaccine. Only 2 febrile seizures occurred after the new procedure was implemented, both occurring in children who did not receive the second acetaminophen suppository. These data were submitted to the European Medicines Agency (EMA), and rare febrile seizures in young children has been added to the undesirable effect section of the Summary Product Characteristics.

Overall, there were 17 cases considered to be related to the J&J vaccine product. This determination was made by the local providers and UMURINZI clinical physicians largely based on participant history and timing of symptom onset soon after vaccination, usually within 24 hours. Ten of these cases were the postvaccination febrile convulsions characterized above. The other 7 (fever, vomiting, and/or diarrhea) also occurred in children <8 years old. These SAEs that were related to vaccination were rare in relation to the total vaccinated, occurring in only 0.041% of children vaccinated.

Details of contraceptive use and predictors of incident pregnancies and outcomes will be presented in a separate manuscript. A randomized clinical trial of safety of this Ebola vaccination regimen in pregnant women and their newborns is under way (ClinicalTrials.gov identifier NCT04556526) [26].

The symptoms reported as UAEs seem to be consistent with previous report and consistent with the approved label. In prior clinical trials, systemic adverse events were also most commonly headache, fatigue, myalgia, arthralgia, and chills; at least 1 of these symptoms occurred in roughly 56%–77% of all adult participants after receiving Ad26.ZEBOV vaccination in those trials [8–15]. While 61% of UAEs described pyrexia, this is still <1% of all vaccinated individuals in the UMURINZI campaign and not more frequent than EMA and WHO summaries of unblinded safety data, which report pyrexia post-Ad26.ZEBOV vaccination in 11%–12% of children and 7% of adults and grade 3 pyrexia in <1.5% of children and 1.4% of adults [16, 27].

This large campaign reaching >200 000 people offers significant additional safety knowledge about the first Ad26.ZEBOV vaccine as part of the heterologous 2-dose Ad26.ZEBOV and MVA-BN-Filo Ebola vaccination regimen. Of particular interest is the identification of rare postvaccination febrile seizures in young children and the decreased incidence of this SAE after the implementation of prophylactic preventive strategies such as acetaminophen. Our findings also highlight the importance of providing contraceptive services when vaccinating women of childbearing age to enhance retention.

## Notes

***Acknowledgments.*** We acknowledge and thank the district health teams and the people who were vaccinated in this vaccination campaign.

***Financial support.*** UMURINZI was co-funded by the Wellcome Trust/UK Foreign, Commonwealth and Development Office (formerly the UK Department for International Development), and J&J. Janssen Vaccines & Prevention B.V. provided financial support and made available the clinical supply of vaccines. Additionally, the J&J Global Public Health team was engaged to provide implementation and community engagement support, as well as health system capacity for UMURINZI. These activities were supported through J&J’s work in the Ebola Vaccine Deployment, Acceptance and Compliance Consortium, which is funded by the Innovative Medicines Initiative, and also includes the London School of Hygiene and Tropical Medicine, World Vision, and the Grameen Foundation. It is to be determined which funding source will pay for the Open Access fees; these charges may be paid by non-grant funds.
